# Nutrient-Regulated Antisense and Intragenic RNAs Modulate a Signal Transduction Pathway in Yeast

**DOI:** 10.1371/journal.pbio.0060326

**Published:** 2008-12-23

**Authors:** Masafumi Nishizawa, Tae Komai, Yuki Katou, Katsuhiko Shirahige, Takehiko Ito, Akio Toh-e

**Affiliations:** 1 Department of Microbiology and Immunology, Keio University School of Medicine, Tokyo, Japan; 2 Department of Material and Biological Sciences, Faculty of Science, Japan Women's University, Tokyo, Japan; 3 Department of Biological Science, Graduate School of Bioscience and Biotechnology, Tokyo Institute of Technology, Yokohama, Japan; 4 Research Center for Advanced Science and Technology, Mitsubishi Research Institute, Tokyo, Japan; 5 Department of Biological Sciences, The University of Tokyo Graduate School of Science, Tokyo, Japan; Howard Hughes Medical Institute Janelia Farm, United States of America

## Abstract

The budding yeast Saccharomyces cerevisiae alters its gene expression profile in response to a change in nutrient availability. The *PHO* system is a well-studied case in the transcriptional regulation responding to nutritional changes in which a set of genes (*PHO* genes) is expressed to activate inorganic phosphate (Pi) metabolism for adaptation to Pi starvation. Pi starvation triggers an inhibition of Pho85 kinase, leading to migration of unphosphorylated Pho4 transcriptional activator into the nucleus and enabling expression of *PHO* genes. When Pi is sufficient, the Pho85 kinase phosphorylates Pho4, thereby excluding it from the nucleus and resulting in repression (i.e., lack of transcription) of *PHO* genes. The Pho85 kinase has a role in various cellular functions other than regulation of the *PHO* system in that Pho85 monitors whether environmental conditions are adequate for cell growth and represses inadequate (untimely) responses in these cellular processes. In contrast, Pho4 appears to activate some genes involved in stress response and is required for G_1_ arrest caused by DNA damage. These facts suggest the antagonistic function of these two players on a more general scale when yeast cells must cope with stress conditions. To explore general involvement of Pho4 in stress response, we tried to identify Pho4-dependent genes by a genome-wide mapping of Pho4 and Rpo21 binding (Rpo21 being the largest subunit of RNA polymerase II) using a yeast tiling array. In the course of this study, we found Pi- and Pho4-regulated intragenic and antisense RNAs that could modulate the Pi signal transduction pathway. Low-Pi signal is transmitted via certain inositol polyphosphate (IP) species (IP_7_) that are synthesized by Vip1 IP_6_ kinase. We have shown that Pho4 activates the transcription of antisense and intragenic RNAs in the *KCS1* locus to down-regulate the Kcs1 activity, another IP_6_ kinase, by producing truncated Kcs1 protein via hybrid formation with the *KCS1* mRNA and translation of the intragenic RNA, thereby enabling Vip1 to utilize more IP_6_ to synthesize IP_7_ functioning in low-Pi signaling. Because Kcs1 also can phosphorylate these IP_7_ species to synthesize IP_8_, reduction in Kcs1 activity can ensure accumulation of the IP_7_ species, leading to further stimulation of low-Pi signaling (i.e., forming a positive feedback loop). We also report that genes apparently not involved in the *PHO* system are regulated by Pho4 either dependent upon or independent of the Pi conditions, and many of the latter genes are involved in stress response. In S. cerevisiae, a large-scale cDNA analysis and mapping of RNA polymerase II binding using a high-resolution tiling array have identified a large number of antisense RNA species whose functions are yet to be clarified. Here we have shown that nutrient-regulated antisense and intragenic RNAs as well as direct regulation of structural gene transcription function in the response to nutrient availability. Our findings also imply that Pho4 is present in the nucleus even under high-Pi conditions to activate or repress transcription, which challenges our current understanding of Pho4 regulation.

## Author Summary

How does a microorganism adapt to changes in its environment? Phosphate metabolism in the budding yeast Saccharomyces cerevisiae serves as a model for investigating mechanisms involved in physiological adaptation. The nutrient inorganic phosphate (Pi) is essential for building nucleic acids and phospholipids; when yeast cells are deprived of Pi, genes required for scavenging the nutrient are activated. This activation is mediated by the Pho4 transcription factor through its migration into or out of nucleus. The Pi-starvation (low-Pi) signal is transmitted by a class of inositol polyphosphate (IP) species, IP_7_, which is synthesized by one of two IP_6_ kinases, Vip1 or Kcs1. However, the IP_7_ made primarily by Vip1 is key in the signaling pathway. Here we report that under Pi starvation Pho4 binds within the coding sequence of *KCS1* to activate transcription of both intragenic and antisense RNAs, resulting in the production of a truncated Kcs1 protein and the likely down-regulation of Kcs1 activity. Consequently Vip1 can produce more IP_7_ to enhance the low-Pi signaling and thus form a positive feedback loop. We have also demonstrated that Pho4 regulates, both positively and negatively, transcription of genes apparently uninvolved in cellular response to Pi starvation and that it sometimes does so independently of Pi conditions. These findings reveal mechanisms that go beyond the currently held model of Pho4 regulation.

## Abstract 3

During nutritional adaptation, transcriptional activation in yeast produces noncoding RNAs that allow the formation of a positive-feedback regulatory loop.

## Introduction

When environmental conditions change, the budding yeast Saccharomyces cerevisiae, like other microorganisms, makes a decision about growth, cell division, and which responses to elicit in a coordinated fashion. Starvation for nutrients, alterations in temperature or salt concentration, and the presence of toxic agents are critical stresses for yeast cells and elicit signals that evoke cellular responses favoring survival under the new conditions. Nutrient status is probably the most important condition that must be accurately and rapidly sensed and responded to in order to ensure cell survival. In this process, nutrient-sensing kinases including cyclic-adenosine-monophosphate-dependent kinase, Snf1**,** Tor, and Pho85 kinases play important roles in regulation at levels ranging from transcription to the activity of individual enzymes [1,2]. Transcriptional regulation is the most fundamental process in the nutritional response, and DNA-binding transcription factors and genes under their control are extensively characterized by conventional genetic and biochemical analyses and, more recently, expression profiling via DNA microarray and chromatin immunoprecipitation (ChIP)-on-chip analysis. The *PHO* system is a well-studied case in which a set of genes (*PHO* genes) is expressed to activate inorganic phosphate (Pi) metabolism for adaptation to Pi starvation [3]. The Pho4 transcription factor that activates *PHO* genes is regulated by phosphorylation to alter its cellular localization: under high-Pi conditions, the Pho85 kinase phosphorylates Pho4, thereby excluding it from the nucleus and resulting in repression (i.e., lack of transcription) of *PHO* genes. Pi starvation triggers an inhibition of Pho85 kinase, leading to the migration of unphosphorylated Pho4 transcriptional activator into the nucleus and enabling expression of *PHO* genes [4–6]. Transcriptional regulation responding to nutrient change is also extensively studied in glucose repression and in amino acid starvation, cases in which a complex interplay between activators and repressors acting on the structural genes involved in the respective process is well documented [7,8].

Recent studies on transcriptional regulation have revealed the participation of novel regulators in addition to protein factors, specifically, an involvement of RNA in the regulation of protein expression responding to external signals including nutrient changes [9,10]. Prokaryotic mRNAs that change their conformation upon binding of specific metabolites can alter transcription elongation or translation initiation and are called riboswitches [11]. Noncoding (nc) RNAs including small inhibitory (si), micro (mi), and small nucleolar (sno) RNAs modify RNA species to regulate gene expression: siRNA and miRNA target mRNA to cause mRNA cleavage and inhibition of translation, respectively, whereas snoRNA targets rRNA. Numerous ncRNAs, however, have been found that do not show these known functions, including antisense (AS) RNAs and transcribed pseudogenes [10]. In S. cerevisiae, several ncRNAs involved in transcriptional regulation are reported: *SRG1* intergenic RNA functions in repression of *SER3* [12,13], and an AS RNA in the *PHO5* locus appears to facilitate *PHO5* transcription upon activation [14], whereas those in *PHO84* and *IME4* function in gene silencing in aging cells [15] and inhibition of transcription [16], respectively (Accession numbers for genes described in this article are listed in Table S1). Recent large-scale cDNA analysis [17,18] and mapping of RNA polymerase II binding using a high-resolution tiling array [19] have identified a large number of intergenic, intragenic, and AS RNA species whose functions are yet to be clarified.

The Pho85 kinase has a role in various cellular functions other than regulation of the *PHO* system via Pho4; these functions include nutrient sensing, cell cycle progression, stress response, and control of cell morphology [20–23]. Pho85 monitors whether environmental conditions are adequate for cell growth and represses inadequate (untimely) responses in these cellular processes [1]. When nutrient is sufficient, the kinase phosphorylates Gsy2, a glycogen synthase [24], and represses the expression of *UGP1*, which encodes an enzyme that catalyzes the production of UDP-glucose for glycogen synthesis [25]. Both of these events lead to the down-regulation of glycogen synthesis. Pho85 also facilitates the degradation of Gcn4, a transcription factor that activates genes involved in amino acid metabolism when amino acids are depleted [26].

In contrast, the known cellular function of Pho4 seems rather limited to the *PHO* system [3]. Recent microarray analysis, however, has demonstrated that some genes involved in stress response and various other metabolic functions are activated under Pi-limiting conditions [27], implying that Pho4 may activate these genes. Indeed, Pho4 is required for G_1_ arrest caused by DNA damage [28]. These observations suggest that Pho4 facilitates stress response by activating genes involved in the process. The fact that overproduction of Pho4 causes growth arrest of yeast cells in the absence of Pho85 [29] supports the antagonistic function of these two players on a more general scale when yeast cells must cope with stress conditions. To explore the general involvement of Pho4 in stress response, we tried to identify Pho4-dependent genes by a genome-wide mapping of Pho4 and Rpo21 binding (Rpo21 being the largest subunit of RNA polymerase II) using a yeast tiling array. In the course of this study, we found that Pho4- and Pi-dependent AS and intragenic RNAs modulate Pi signaling, leading to stimulation of expression of *PHO* genes, which demonstrates that nutrient-regulated RNA species other than mRNA are functioning in nutrient-responsive pathways in yeast cells. We also found that Pho4 was involved in transcriptional regulation of stress-responsive genes, either positively or negatively, and in some cases independently of environmental Pi conditions, which challenges the current model of Pho4 regulation [4,5].

## Results

### ChIP-on-Chip Analysis Revealed Novel *PHO*-Type Genes

**Figure 1:**
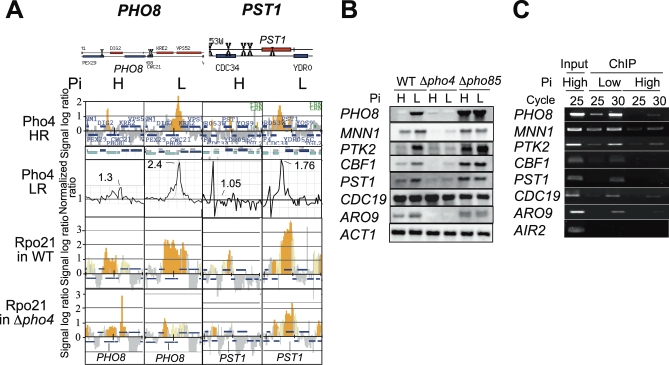
Phosphate Condition-Dependent Pho4 Binding and Pho4-Dependent Expression of Genes (A) Localization of Pho4 around the *PHO8* and *PST1* loci. Schematic representations of the regions of these genes (adopted from the results of pattern matching analysis provided by Saccharomyces Genome Database [SGD], http://www.yeastgenome.org/ATContents.shtml) showing prospective Pho4 binding sites (X) are at the top. The next two rows show Pho4 localization to these regions in the wild-type (WT) cells grown under high (H)- or low (L)-phosphate (Pi) conditions analyzed with high- and low-resolution (HR and LR) chips. Localization of Rpo21, the largest subunit of RNA polymerase II, to these regions in the WT or a Δ*pho4* mutant analyzed with HR chip is shown in the bottom two rows. Yeast cells containing Pho4-Flag × 3 or Rpo21-Flag × 3 were processed for chromatin immunoprecipitation (ChIP) as described in Methods, and enrichment in the immunoprecipitated fraction relative to whole genome DNA is shown in each panel. Blue bars above and below the *x*-axis represent genes on the Watson and Crick strands, respectively. The *y*-axis scales are log_2_(signal ratio) and normalized ratio for HR and LR chips, respectively. In HR chip panels, brown bars represent signals that are judged as significant binding, whereas gray bars are not. The normalized ratio values of the peaks are shown in the LR chip panels. (B) Northern analysis of genes showing Pho4- and Pi-dependent expression. Total RNA was isolated from the wt, Δ*pho4*, or Δ*pho85* cells grown under high (H)- or low (L)-Pi conditions, subjected to northern blot analysis, and probed with a digoxigenin (DIG)-labeled probe for each gene as designated. (C) Enrichment of promoter regions in the chromatin-immunoprecipitated (ChIPed) DNA fragments dependent on Pi conditions analyzed by gene-specific PCR. Total DNA in the whole cell extract (input) or ChIPed DNA fragments prepared from the wt cells grown under high- or low-Pi conditions were amplified by PCR for 25 or 30 cycles using primer pairs specific to the promoter regions of the genes designated on the left-hand side of the panel. The *AIR2* ORF is the negative control for Pho4 binding.

To analyze the Pho4 binding sites in the yeast genome, we used two kinds of oligo-DNA arrays, an Affymetrix high-density oligo-DNA array harboring 25-mer oligonucleotides with 4-nucleotide spatial resolution (high-resolution [HR] chip) and an Agilent yeast whole genome 44K array that had 60-mer oligos with ca. 270-nucleotide spatial resolution (low resolution [LR] chip). The complete datasets of the HR chip analysis are found in the NCBI GEO database (http://www.ncbi.nlm.nih.gov/geo/) under accession number GSE13350. Analysis with the HR and LR arrays revealed that Pho4 bound to 51 and 57 genes, respectively, under low- but not high-Pi conditions (Table S2). Thirty-five genes were common in the two analyses, and all but two of these (*KCS1* and *SHE9*) had a prospective Pho4 binding sequence (CACGTG/T or CTGCAC) in their upstream regions. For the two exceptions, a binding sequence was present within the ORF (Table S2). Among the 35 genes, 16 had already been reported as *PHO* genes, that is, genes regulated by Pho4 in a Pi-dependent manner, by genetic, biochemical, and microarray analyses [30,31]. Our results demonstrated that Pho4 actually binds to the upstream regions of these genes depending on Pi conditions. In addition to the known *PHO* genes, our analyses identified 19 genes as possible novel *PHO*-type genes. Among them, eight genes, *PST1*, *MNN1*, *HOM3*, *HOR7*, *PTK2*, *CBF1*, *SUR1*, and *GLN1*, showed the expected pattern of Rpo21-binding (Table S2). Representative results for *PHO8* and *PST1* are shown in Figure 1A: Pho4 binding to the upstream regions of these two genes depending upon Pi condition is demonstrated by the two different ChIP-on-chip analyses (Figure 1A), Pi- and Pho4-dependent transcription by Rpo21 binding (Figure 1A) and by northern analysis (Figure 1B), and a Pi-dependent in vivo binding of Pho4 to their promoter regions by gene-specific PCR of a chromatin-immunoprecipitated (ChIPed) fragment (Figure 1C). Pi- and Pho4-dependent transcription of *MNN1*, *PTK2*, *CBF1*, and *PST1* was demonstrated by northern analysis (Figure 1B). In vivo binding of Pho4 to the upstream regions of these genes depending on Pi conditions was demonstrated by PCR using ChIPed DNA as a template and primers specific to the respective gene, which was in good accordance with the results of ChIP-on-chip analysis (Figure 1C and Table S2). *AIR2* serves as a negative control for Pho4 binding, because it has no prospective Pho4 binding sites in its ORF and its expression is not affected by Pi conditions or Pho4. *ARO9* shares a divergent promoter with *SPL2*, a known *PHO*-gene. Expression of *ARO9* appeared dependent on both Pi and Pho4 (Figure 1B), and Pho4 bound to its promoter in a Pi-dependent fashion (Figure 1C). Binding of Pho4 to the *CYC3*-*CDC19* region appeared to be Pi-dependent (Table S2), but *CDC19* expression appeared independent of Pi conditions or Pho4 (Figure 1B and 1C), indicating that Pho4 binding to the upstream region of *CDC19* did not play a major role in transcriptional regulation of *CDC19*.

### Pho4 Can Either Activate or Repress Gene Expression Independent of Phosphate Conditions

**Figure 2:**
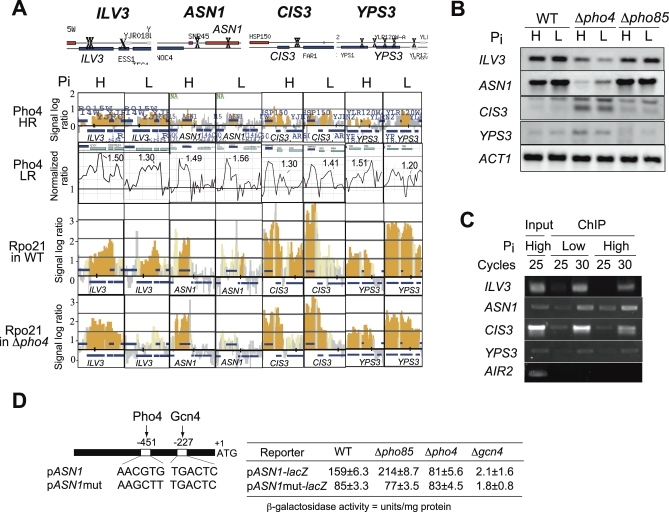
Phosphate-Independent Localization of Pho4 to the Intergenic Region or ORF of Yeast Genes (A) Localization of Pho4 around the *ILV3*, *ASN1*, *CIS3*, and *YPS3* loci. Prospective Pho4 binding sites (X) in each region are shown in the schematic representation of the regions containing these genes at the top. Next two rows show Pho4 localization to these regions in the wild-type (WT) cells grown under high (H)- or low (L)-phosphate (Pi) conditions analyzed with high- and low-resolution (HR and LR) chips. The bottom two rows show Rpo21 localization to these regions analyzed with an HR chip. (B) Northern analysis of genes showing Pi-independent but Pho4-dependent expression, as in the legend to Figure 1B. Pho4 dependency was either positive (*ILV3* and *ASN1*) or negative (*CIS3* and *YPS3*) for expression. *ACT1* is the loading control. (C) Enrichment of promoter regions in the ChIPed DNA fragments independent of Pi conditions analyzed by gene-specific PCR. Conditions for PCR are as described in the legend to Figure 1. (D) A schematic representation of the *ASN1* promoter showing Pho4 and Gcn4 binding sites (AACGTG and TGACTC, respectively) and activities of the wt and mutant (*ASN1*mut; lacking Pho4 binding site) promoters represented by β-galactosidase activity. The wt, Δ*pho85*, Δ*pho4*, or Δ*gcn4* cells harboring respective reporter plasmids were grown to the mid-log phase under high-Pi conditions before the preparation of cell extracts. The values are an average of three different assays, each of which contains measurements with three independent clones.

ChIP-on-chip analyses with the HR and LR arrays revealed that 140 and 30 genes, respectively, showed Pi-independent binding of Pho4, and among them, nine genes (*URA3*, *MUC1*, *CIS3*, *ILV3*, *PDC1*, *YPS3*, *YLR137W*, *HPF1*, and *ASN1*) were commonly detected. Each of these nine genes has the prospective Pho4 binding site in its promoter or ORF (Table S3). We focused on *ILV3*, *ASN1*, *CIS3*, and *YPS3* for further analysis. Analyses of the binding profiles of Pho4 to these genes by ChIP-on-chip analysis using the two different platforms are shown in Figure 2A. Although the binding profiles of Rpo21 in the wild-type (wt) or Δ*pho4* mutant exhibited some inconsistency with those expected from Pho4 binding profiles, northern analysis demonstrated Pi-independent but Pho4-dependent expression, either a decrease (*ILV3* and *ASN1*) or an increase (*CIS3* and *YPS3*) in the absence of Pho4 (Figure 2B). Gene-specific PCR demonstrated that Pho4 bound to the upstream region of these genes in vivo irrespective of Pi conditions (Figure 2C). These results raise possibilities that Pho4 can bind to a promoter under high-Pi conditions and that Pho4 binding can lead to transcriptional activation (*ASN1* and *ILV3*) or repression (*CIS3* and *YPS3*). The former hypothesis challenges the current model of Pho4 regulation in which, under high-Pi conditions, Pho4 is excluded from the nucleus through phosphorylation by Pho85-Pho80 [5]. Therefore, we further analyzed whether the activity of the *ASN1* promoter was dependent on Pho4 under high-Pi conditions by measuring reporter activity in cells grown in a high-Pi medium. The wt promoter was active under high-Pi conditions (Figure 2D), and its activity level decreased to almost 50% when a prospective Pho4 binding site (at −451 with A of ATG as +1) in the promoter was mutated (*ASN1*mut). In the absence of Pho85 where Pho4 became active, the activity of the wt promoter was stimulated by 1.3-fold compared to that in the wt strain, but the *ASN1*mut promoter showed a level of the activity similar to that observed in the wt strain. In the absence of Pho4 (Δ*pho4*), the activity of the wt *ASN1* promoter decreased to 50% whereas that of the mutant promoter showed a similar level of the activity in the wt cells. Gcn4 activates *ASN1* under amino-acid-starvation conditions [32], and in the absence of Gcn4 (Δ*gcn4*), the promoter activity was decreased drastically (Figure 2D). These results further demonstrated that Pho4 can activate *ASN1* regardless of Pi conditions, which requires the Pho4 binding sequences in its promoter.

### Phosphate- and Pho4-Regulated Antisense and Intragenic RNAs in the *KCS1* Locus

**Figure 3:**
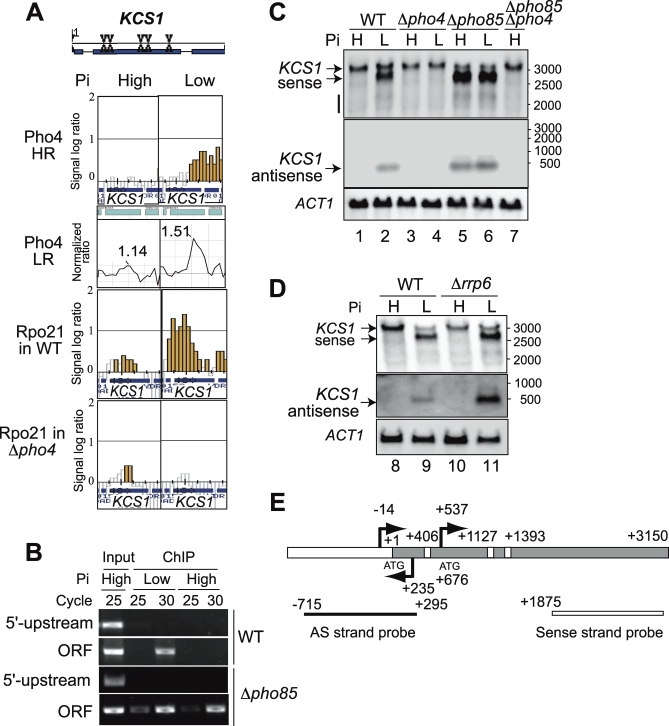
Pi- and Pho4-Dependent Antisense and Intragenic RNA Species in the *KCS1* Locus (A) The positions of possible Pho4 binding sites within the *KCS1* ORF (X) and Pi-dependent localization of Pho4 within the *KCS1* ORF detected by HR- and LR-chip analyses are shown in the top two panels. In this case, the results using an HR chip bearing chromosome 3, 4, 5, and 6 sequences are shown for better resolution. Pi- and Pho4-dependent localization of Rpo21 to the *KCS1* locus in the wt and Δ*pho4* strains under high- or low-Pi conditions using the HR chip is shown in the bottom two panels. The slight difference in the data processing programs between the chromosome 3–6 chip and the whole genome chip caused the different appearance of the figures as compared to Figures 1A and 2A. (B) Demonstration of Pi-dependent enrichment of the *KCS1* ORF fragment but not the 5′-upstream region by gene-specific PCR. Total DNA in the whole cell extract (input) or ChIPed DNA fragments prepared from the wt or Δ*pho85* cells grown under high- or low-Pi conditions were subjected to PCR using the primer set specific to the 5′-upstream region or ORF of *KCS1* as designated. The conditions for PCR are the same as those described in the legend to Figure 1. (C) Northern analysis of *KCS1* sense and AS RNA expression in the wt and various *pho* mutant strains as designated under high (H)- or low (L)-Pi conditions using strand-specific DIG-RNA probes. For detection of *KCS1* sense transcripts, an RNA probe specific to the 3′ region of *KCS1* was employed (see Figure 3E). The arrows on the left-hand side of the panel indicate the positions of the *KCS1* sense (the top panel) and AS transcripts (the middle panel), respectively. The vertical bar on the left-hand side of the top panel designates short *KCS1* sense transcripts ranging from 2,300 to 1,800 nt. The positions of RNA size markers are shown on the right. *ACT1* is the loading control. (D) Effect of a Δ*rrp6* mutation on the levels of the *KCS1* sense and AS RNA expression. Total RNA was isolated from the wt or Δ*rrp6* mutant cells grown under different Pi conditions as described above and subjected to northern analysis using strand-specific DIG-RNA probes as designated. The positions of RNA size markers are shown on the right. *ACT1* is the loading control. (E) A schematic representation of the structure of the *KCS1* gene showing the positions of the ATG codon at +1 and +676, taking A of the initiating ATG as +1. The blank bar represents the promoter region, and the shaded one is the coding region. The white boxes in the coding region are the prospective Pho4 binding sites at +406, +1127, and +1393. The arrows on the bar designate the major transcription start points of the sense RNA in the 5′-upstream region (at −14) and in the ORF (at +537), and that below the bar is that of the AS RNA (+235), determined by the 5′-RACE method. The boxes below the bar indicate the positions of the strand-specific probes used for northern analysis: the blank box is a sense-strand-specific probe (+3150 to +1875), and the black one is the AS strand (−715 to +295).

Our ChIP-on-chip analyses using two different platforms demonstrated the binding of Pho4 within the *KCS1* and *SHE9* ORFs depending on Pi conditions (Figure 3A and Table S2). Because, to our knowledge, gene transcription mediated by the binding of a yeast transcription factor within an ORF is very rare with only three precedents [33–35], we further analyzed the regulation of *KCS1* by Pho4. Whereas gene-specific PCR using a primer set specific to the *KCS1* promoter (−908 to +70) failed to detect an enrichment of the Pho4-bound fragment (Figure 3B, top panel), the ORF-specific primer (+36 to +1102) could detect an enrichment of the Pho4-bound fragment prepared only from cells grown in low-Pi medium (Figure 3B, second panel from the top). In the absence of Pho85 where Pho4 became active, Pho4 binding to the ORF was detected under both high- and low-Pi conditions (Figure 3B, bottom panel) whereas that to the upstream region was not (Figure 3B, second panel from the bottom). These results demonstrated that Pho4 binding within the *KCS1* ORF depends on Pi conditions. We also analyzed Rpo21 binding by ChIP-on-chip and found that Rpo21 was localized to the *KCS1* locus in Pi- and Pho4-dependent manners (Figure 3A, bottom two panels). This result indicated the presence of Pi- and Pho4-dependent transcription in the *KCS1* locus. To examine whether binding of Pho4 within the ORF could direct *KCS1* mRNA transcription, initiation of transcription within the ORF (intragenic RNA), or synthesis of AS RNA, we carried out northern analysis using RNA probes specific to the sense or antisense strands of *KCS1* (Figure 3C). With an RNA probe hybridizing with the *KCS1* mRNA (Figure 3E), a transcript of ca. 3,200 nucleotides (nt), approximately the size of the *KCS1* ORF (3,150 nt; Figure 3E), was detected, which was not dependent on either Pho4 or Pi conditions (Figure 3C, top panel, lanes 1 to 4, designated by upper arrow). Judging from its size, this RNA species is highly likely to be the *KCS1* mRNA, because this band was not detected in Δ*kcs1* cells (unpublished data). In the absence of Pho85, a transcript with a smaller size (ca. 2,600 nt) was detected (Figure 3C, top panel, lanes 5 and 6, designated by lower arrow), together with the *KCS1* mRNA and short transcripts ranging from 2,300 to 1,800 nt in length (lanes 2, 5, and 6, designated by a vertical bar). These short sense transcripts were also detected in the wt cells under low-Pi conditions, though weakly (lane 2), but not observed in a Δ*pho4* (lanes 3 and 4) or Δ*pho4* Δ*pho85* double mutant (lane 7). These results indicated that Pho4 binding within the *KCS1* ORF can activate the transcription of RNAs shorter than the *KCS1* mRNA in both Pi- and Pho4-dependent manners. It should be noted that the *KCS1* mRNA level appeared reduced when these short RNA species were abundant in Δ*pho85* cells (lanes 5 and 6). On the other hand, an AS RNA probe covering from −715 to +295 of the *KCS1* gene (Figure 3E) could detect a short transcript of ca. 500 nt, which was dependent on both Pho4 and Pi conditions (Figure 3C, middle panel, lanes 1 to 7), whereas that covering from −715 to −228 failed to do so (unpublished data). Therefore, the AS RNA was highly likely to be encoded between +295 and −228. The presence of AS RNA in *PHO* genes is reported in *PHO5* [14] and *PHO84* [15], and in both cases, *RRP6* affects the stability of the AS RNA. We tested the effect of a Δ*rrp6* mutation and found that the mutation also stabilized the AS RNA in the *KCS1* locus while not significantly affecting the amount of sense RNAs (Figure 3D).

To examine whether the 2,600 nt transcript is a processing product and to confirm the presence of the AS RNA, we determined transcriptional start points of the sense and AS RNAs in the Δ*pho85* strain by the 5′ rapid amplification of cDNA ends (RACE) method and found that the sense RNAs started mainly at −14 and +537 and that the AS transcription started at +235 (Figure 3E). The sense RNA starting at −14 is highly likely to be the *KCS1* mRNA, because the transcription start points of the sense RNA in the wt were also mapped mainly to this point (unpublished data). The one starting at +537 can be ca. 2,600 nt in length when transcribed through the ORF, the size of which coincides well with the estimated size of the short transcript detected by northern analysis (Figure 3C, top panel, designated by lower arrow). This result supported the conclusion that the 2,600 nt RNA is not a processing product but is transcribed from within the *KCS1* coding sequence.

**Figure 4:**
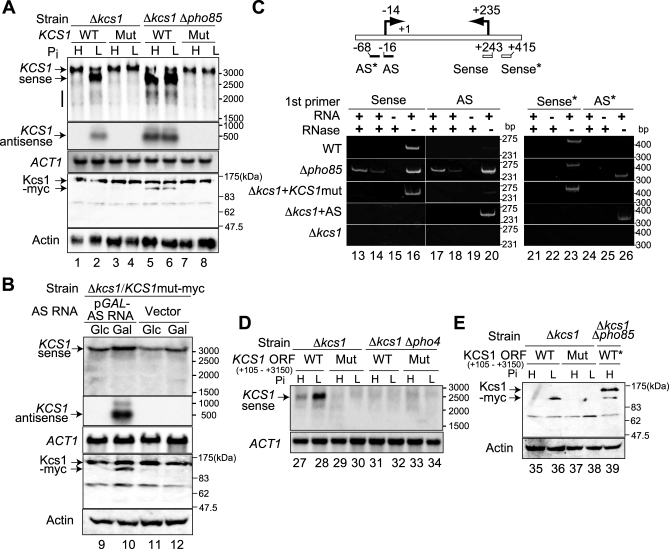
Requirement of Pho4 Binding Sites for the Expression of the *KCS1* Intragenic and AS RNAs and for the Production of the Truncated Kcs1 Protein (A) Expression of the *KCS1* sense and AS RNAs from the wt or mutant (Mut; lacking the three Pho4 binding sites) *KCS1* gene in a YCp plasmid in Δ*kcs1* or Δ*kcs1* Δ*pho85* strain under high (H)- or low (L)-Pi conditions was analyzed by northern blotting with strand-specific RNA probes. Positions of the *KCS1* sense (top panel) and AS RNA (second panel from the top) are designated by arrows and a vertical bar on the left-hand side of the panels. The positions of RNA size markers (nt) are on the right. *ACT1* is the loading control. The Kcs1 protein tagged with the c-*myc* epitope at its C terminus was detected by western blotting using anti-*myc* antibody. The full-length and truncated Kcs1 proteins are designated by two arrows on the left-hand side of the second panel from the bottom. The positions of marker proteins are on the right. Actin is the loading control for western analysis. The faint band between 83 and 62 kDa markers observed in all lanes is nonspecific staining of yeast protein by the anti-*myc* antibody. (B) Heterologous *KCS1* AS RNA expression and production of the truncated Kcs1 protein. A Δ*kcs1* strain harboring *KCS1*mut-*myc* in a YCp vector and p*GAL1*-AS RNA in a YEp vector or vector alone was analyzed for *KCS1* sense and AS RNA expression by northern blotting as designated. AS RNA expression was induced by incubating the transformants in galactose medium for 6 h (Gal) or repressed in glucose medium (Glc). Production of Kcs1-*myc* protein was detected as described above. (C) An RNA hybrid formation of the *KCS1* mRNA with the AS RNA detected by RNase protection assay and RT-PCR. A schematic representation of the *KCS1* gene of interest is shown at the top. The arrows on the bar designate the major transcription start points of the mRNA (at −14) and of the AS RNA (+235). The boxes below the bar indicate the positions of the strand-specific primers used for the detection of dsRNA: the blank boxes are sense-strand-specific primers (sense, +243 to +223, and sense*, +415 to +395), and the black ones are primers for the AS strand (AS, −16 to +5, and AS*, −68 to −48). Total RNA was isolated from the wt, Δ*pho85*, Δ*kcs1*, Δ*kcs1* harboring *KCS1*mut, or Δ*kcs1* expressing the AS RNA under the *TDH3* promoter, grown in high-Pi medium, except that Δ*kcs1* harboring *KCS1*mut was in low-Pi medium. About 10 μg of each RNA sample was incubated with (+) or without (–) RNase ONE. For negative control (–RNA), total RNA was replaced by yeast tRNA. The digestion products were then used as templates for first-strand cDNA synthesis with strand-specific primers as designated, and the cDNA products were subjected to PCR amplification with the appropriate reverse primer. The sense-strand-specific (sense) primer, from +243 to +223; antisense-strand-specific (AS), from −16 to +5; sense*, from +415 to +395; and AS*, from −68 to −48. For lanes 14, 16, 18, 20, 23, and 26, one-tenth of the amount of the cDNA template was used for PCR compared to those in the other lanes. The PCR products were analyzed by electrophoresis on a 5% polyacrylamide gel, and 10-fold dilutions of the PCR products were loaded in lanes 16, 20, 23, and 26. The positions of DNA size markers are shown on the right-hand side of each panel. (D) Pho4-directed and Pi-dependent transcription within the *KCS1* ORF. The N-terminally truncated wt or mutant (Mut) *KCS1* ORF (+105 to +3150) placed in a YCp plasmid was introduced into Δ*kcs1* or Δ*kcs1* Δ*pho4* strain, and transcription of sense RNA under high (H)- or low (L)-Pi conditions was detected by northern blotting using a strand-specific probe (top panel). The positions of RNA size markers are shown on the right and *ACT1* is the loading control. (E) The intragenic RNA also can encode the truncated Kcs1-*myc* protein. The truncated wt or Mut *KCS1* ORF (+105 to +3150) tagged with c-*myc* at its C terminus was introduced into the Δ*kcs1* strain, and the production of Kcs1-*myc* protein was analyzed by western blotting. WT* designates the full-length wt *KCS1* gene tagged with c-*myc*, which produced the normal and truncated Kcs1-*myc* protein (lane 39) as in lane 5 of Figure 4A. Actin is the loading control for western analysis.

Three prospective Pho4 binding sites (at +406, +1127, and +1193) are present in the 5′ half of the *KCS1* ORF (Figure 3E), and the one closest to the 5′ end is sandwiched by the transcription start points of the intragenic (+537) and AS RNAs (+235), which suggests a possibility that binding of Pho4 to the +406 site activates both of the 2,600 nt sense and AS RNAs. We constructed the *KCS1* mutant (*KCS1*mut) in which three prospective Pho4 binding sites were mutated while keeping the amino acid sequence intact and analyzed the wt or *KCS1*mut in a low copy (YCp) plasmid in Δ*kcs1* cells. We found that the *KCS1* mRNA was normally produced (Figure 4A, top panel, lanes 1 to 4), whereas the AS and intragenic RNAs were produced from wt *KCS1* but not from *KCS1*mut under low-Pi conditions (second panel, lanes 2 and 4). Both the AS and truncated sense RNAs were synthesized from wt *KCS1* in Δ*kcs1* Δ*pho85* cells (lanes 5 and 6) whereas *KCS1*mut produced RNA of the wt size but not the intragenic or AS RNAs (lanes 7 and 8). These results indicated that the generation of the 2,600 nt intragenic as well as AS RNAs are activated by Pho4, which requires the presence of at least one of the prospective Pho4 binding sites. Short sense transcripts around 2,000 nt (Figure 4A, lanes 2, 5, and 6, designated by a vertical bar) decreased to below detectable levels with *KCS1*mut (lanes 7 and 8), which suggested that these transcripts are also dependent on Pho4.

### Antisense RNA Can Cause Production of Truncated Kcs1 Protein

Antisense RNA functions *in cis* to inhibit sense RNA transcription by transcriptional collision as reported in the *IME4* case [16] and *in trans* to form a hybrid with sense RNA to inhibit its function [9,10]. To reveal the role of the *KCS1* AS RNA that is regulated by Pho4 and Pi conditions, we first asked whether the AS RNA could affect the synthesis of the Kcs1 protein by immunoblotting. Kcs1 protein whose C-terminus was tagged with the c-*myc* epitope was produced from the wt *KCS1* or *KCS1*mut gene in a YCp plasmid (Figure 4A, second panel from the bottom). Kcs1 protein of the wt size (1,143 amino acids including the *myc* tag) was detected in all cases (lanes 1 to 8), and Δ*kcs1* Δ*pho85* cells harboring the wt *KCS1* plasmid produced a truncated protein together with the normal one (Figure 4A, second panel from the bottom, lanes 5 and 6). The band observed between 83 and 62 kDa markers was nonspecific staining with anti-*myc* antibody because it was also detected in the absence of Kcs1-*myc* protein in the extract (unpublished data). Because both the AS and truncated sense RNAs were produced in Δ*kcs1* Δ*pho85* cells, the AS RNA hybridizing to the 5′ region of the *KCS1* mRNA might inhibit normal translation initiation or the truncated sense RNA might be translated, either of which could use the in-frame initiation codon at +676 (Figure 3E) to produce truncated Kcs1 protein composed of 825 amino acids plus 93 amino acids from the c-*myc* tag. To examine whether the AS RNA could act in *trans*, for example, by hybridizing to the 5′ region of the *KCS1* mRNA, we constructed a plasmid in which the AS RNA was produced from the *GAL1* promoter by placing a *KCS1* fragment covering +295 to −950 downstream of the promoter and introduced it into a Δ*kcs1* strain harboring the *KCS1*mut gene, so that the AS RNA was provided only *in trans* and the short sense transcripts including the 2,600 nt intragenic RNA were not produced (Figure 4A, top panel, lane 7). The truncated Kcs1 protein was observed only when transcription of the AS RNA was induced from the plasmid in galactose medium (Figure 4B, second panel from the bottom, lanes 9 and 10). As expected, the short sense transcripts were not detected when the AS RNA was overproduced in *trans* (Figure 4B, top panel). These results indicated that the AS RNA can act *in trans* (i.e., inhibition of the normal translation initiation, possibly by hybrid formation with the *KCS1* mRNA). To test hybrid formation, we tried to detect the presence of the double-stranded (ds) RNA in the total RNA sample by RNase protection analysis using single-stranded RNA-specific RNase, followed by reverse transcription (RT) and PCR amplification of the protected fragments. Reverse transcription was carried out using either sense- or antisense-strand-specific primers, hybridizing to from +243 to +223 and from −16 to +5 (sense and AS in Figure 4C, respectively). As shown in Figure 4C, dsRNA of ca. 250 bp in length protected from RNase digestion was detected in Δ*pho85* cells in which both sense and AS RNAs were present (Figure 4C, second panel from the top, lanes 13, 14, 17, and 18). When the RNA sample was not digested with RNase, the sense strand was successfully amplified, whereas the AS strand was not (lanes 16 and 20), indicating that the *KCS1* mRNA was present but the AS RNA was not in the RNA sample tested. In Δ*pho85* cells in which both sense and AS RNAs were present (Figure 4C, lane 14), dsRNA of ca. 260 bp in length protected from RNase digestion was detected (Figure 4C, second panel from the top, lanes 13, 14, 17, and 18). The Δ*kcs1* mutant cells do not produce the *KCS1* sense or AS RNAs, and accordingly the sense or AS primer failed to synthesize cDNA (Figure 4C, bottom panel). When *KCS1*mut was expressed in Δ*kcs1* cells under low-Pi conditions, the sense RNA was produced but not the AS (Figure 4A, lane 4; Figure 4C, the middle panel, lanes 16 and 20), and therefore dsRNA was not detected (lanes 13, 14, 17, and 18). Reciprocally, when the AS RNA was expressed in Δ*kcs1* cells, only the AS was detected (Figure 4C, second panel from the bottom, lanes 16 and 20), and the protected dsRNA fragments were, if any, below the detectable level (lanes 13, 14, 17, and 18). To confirm that the protected dsRNA was specifically amplified, we carried out the RT reaction using primers hybridizing upstream of the transcription start points of the sense or AS RNAs (at −14 and +235, respectively, in Figure 3E, and those designated by asterisks in Figure 4C). The RNA samples prepared from the strains producing the *KCS1* mRNA (wt, Δ*pho85*, and Δ*kcs1* + *KCS1*mut) could generate cDNA of ca. 430 bp in length when amplified with the sense*/AS primer pair only in the absence of RNase digestion (Figure 4C, lanes 21 and 23). Similarly, the AS*/sense primer pair could synthesize cDNA when the RNA samples containing the AS RNA were not digested by RNase (lanes 24 and 26). These results indicated that the protected dsRNA fragment was specifically transcribed and amplified by the RT-PCR reaction and therefore strongly suggested that the AS RNA can form a hybrid with the *KCS1* mRNA in vivo. Such a hybrid may inhibit normal translation initiation, leading to the generation of the truncated Kcs1 protein.

### Intragenic RNA Can Produce the Truncated Kcs1 Protein

Although we demonstrated that the truncated Kcs1 protein could be generated independently of the short sense RNAs, it is still possible that the truncated protein is translated from the 2,600 nt intragenic RNA. To test this possibility, we constructed plasmids harboring N-terminally truncated *KCS1* or *KCS1*mut ORF fragments (+105 to +3150) and introduced them into Δ*kcs1* or Δ*kcs1* Δ*pho4* mutants. Because these *KCS1* fragments lack the *KCS1* promoter, the plasmids are unable to produce the full-length *KCS1* mRNA. With a strand-specific RNA probe (Figure 3E), we could detect Pi- and Pho4-dependent transcripts of ca. 2,600 nt in length (Figure 4D, lanes 27, 28, 31, and 32). This transcript was not observed in the *KCS1*mut (lanes 29, 30, 33, and 34), indicating that the presence of the Pho4 binding sites is required for the production of this RNA species. Thus Pho4 could activate transcription from downstream of its binding site in the *KCS1* ORF. Although this downstream transcription was plasmid-borne, the observed similarity in the size of transcript and its regulation strongly indicate that the 2,600 nt transcript is actually transcribed in the chromosomal *KCS1* locus in a Pho4-dependent fashion and not a processing (or limited degradation) product. This plasmid-derived transcript could produce protein that had a size similar to that of the truncated Kcs1 protein (Figure 4E, lanes 36 and 39), indicating that the 2,600 nt intragenic RNA can encode the truncated Kcs1 protein. Taken together, these results indicated that Pho4 binding within the *KCS1* ORF provokes transcription of both of the AS and the 2,600 nt intragenic RNAs, which may lead to production of the truncated Kcs1 protein by alteration of translation initiation through the formation of a hybrid with the *KCS1* mRNA and by translation of the 2,600 nt intragenic transcript.

### Pho4 Modulates Phosphate-Signaling Pathway via Antisense and Intragenic RNAs

**Figure 5:**
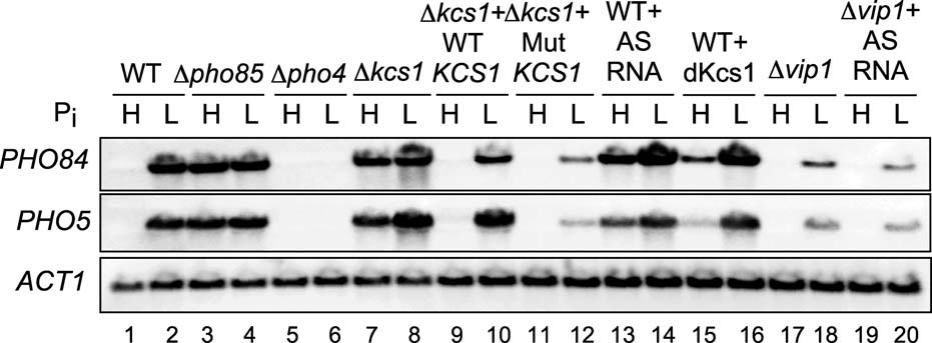
Effect of *KCS1* and *VIP1* on *PHO84* and *PHO5* Expression Analyzed by Northern Blotting The wt, various mutants, or those harboring plasmids expressing *KCS1*, *KCS1*mut, AS RNA, or a truncated Kcs1 protein (dKcs1) were incubated for 5 h in high (H)- or low (L)-Pi media before isolation of total RNA as described in Methods. *ACT1* is the loading control.

What is the biological relevance of Pi- and Pho4-dependent production of the AS and intragenic RNAs and consequently of the truncated Kcs1 protein? *KCS1* codes for inositol hexakisphosphate (IP_6_) kinase synthesizing 5-diphospho myoinositol pentaphosphate (5-PP-IP_5_) [36]. The same substrate is used by another yeast IP_6_ kinase, Vip1, to synthesize IP_7_ isomers, 4- or 6-PP-IP_5_ that function in Pi signaling in the *PHO* system [37,38]. Therefore, it is conceivable that a decrease in the Kcs1 activity can supply more substrate for Vip1, thereby enhancing Pi signaling. The fact that overproduction of Kcs1 reduces the extent of *PHO5* derepression whereas a deletion of *KCS1* derepresses *PHO5* under high-Pi conditions [39] supports this model. Although the levels of normal Kcs1 protein did not appear to be altered significantly in the presence of the AS RNA and 2,600 nt intragenic transcript (Figure 4A), the formation of a hybrid RNA could affect the normal level of the *KCS1* mRNA, which may cause a slight difference in the level of the Kcs1 protein not detectable by western analysis. In addition, the presence of the truncated Kcs1 may perturb normal function of Kcs1. To test these hypotheses, we analyzed the effects of the AS RNA and the intragenic sense transcript on *PHO84* and *PHO5* expression by northern analysis (Figure 5). *PHO84* responds more quickly to a change in Pi conditions than *PHO5* [40]. In the wt cells, *PHO5* and *PHO84* were expressed only under low-Pi conditions (Figure 5, lanes 1 and 2) but not in the absence of Pho4 (lanes 5 and 6). The two genes were expressed under high-Pi conditions in Δ*kcs1* cells (Figure 5, lanes 7 and 8) as reported [39], which was suppressed by the wt *KCS1* in a YCp plasmid (lanes 9 and 10). This result indicated that the plasmid-borne Kcs1 is functional. *KCS1*mut that produced neither the AS RNA nor the intragenic transcript (Figure 4A) showed a decreased expression level of the two genes under low-Pi conditions (Figure 5, lane 12), suggesting that the low-Pi signal was not transmitted sufficiently to activate Pho4. On the other hand, overexpression of the AS RNA in the wt cells resulted in a significant derepression of the two genes under high-Pi conditions (lanes 1 and 13), suggesting that the low-Pi signal was transmitted to activate Pho4 under high-Pi conditions in this case. This stimulatory function of the AS RNA was dependent on the presence of Vip1 IP_6_ kinase (lane 19) that functions in low-Pi signal transmission [38]. This result further supported the conclusion that the AS RNA functions in the low-Pi signal transduction pathway. We also overproduced a truncated Kcs1 protein (dKcs1, +670 to +3150) in the wt cells, which caused derepression of *PHO84*, albeit weakly, and barely detectable expression of *PHO5* under high-Pi conditions (lane 15). The different expression levels of *PHO84* and *PHO5* can be attributed to different responsiveness of the two genes against the change in the environmental Pi level [40]. We also assayed the activity of acid phosphatase encoded by *PHO5* in the strains with a combination of various plasmids as tested in northern analysis and found that the levels of the enzyme activities correlated with the mRNA level (unpublished data). These results indicated that the presence or absence of the AS RNA and the 2,600 nt intragenic RNA cause altered regulation of *PHO5* and *PHO84* responding to Pi conditions, and therefore it is likely that the Kcs1 activity was modulated by the RNAs, the truncated Kcs1 protein, or both. The apparently weak effect on Pi signaling of dKcs1 compared to that of the AS RNA suggests that the truncated protein is not solely responsible for the stimulation of the low-Pi signaling. The AS RNA could play a certain role in this stimulation process, possibly through modulation of the *KCS1* mRNA and protein levels. Thus, Pho4 appears to enhance low-Pi signaling by expressing the AS and the intragenic RNAs from within the *KCS1* ORF, thereby constituting the positive feedback loop in the Pi signaling pathway.

## Discussion

In this paper, we reported three novel findings derived from the ChIP-on-chip analyses of Pho4 and Rpo21 binding throughout the entire yeast genome: (i) the finding of novel *PHO*-type genes, (ii) the ability of Pho4 either to activate or to repress transcription independently of environmental Pi conditions, and (iii) the presence of Pi-regulated AS and intragenic RNAs that modulate Pi signal transmission. We demonstrated that 18 genes that had not been classified previously as involved in the *PHO* system showed Pho4 binding in a Pi-dependent fashion (Table S2 and Figure 1), and at least four of them, viz., *MNN1*, *CBF1*, *PST1*, and *PTK2*, clearly showed Pho4 binding to their promoters in vivo dependent on Pi conditions and consequently transcription that was dependent on both Pi conditions and Pho4 (Figure 1B and 1C). Harbison et al. reported Pho4 binding profiles under low-Pi conditions [41], and their results share *MNN1* and *PTK2* out of our 18 novel *PHO*-type genes. Gonze et al. predicted *ARO9* and *PST1* as *PHO*-type genes by computational analysis [42]. *KCS1* expression is reported to increase under Pi-limiting conditions [27] and in the absence of Pho85 by microarray analysis [43], probably because of the use of an oligo-DNA array bearing 3′-nested probes that detects Pi- and Pho4-dependent intragenic RNA. Cross-regulation of phosphate and sulfate metabolism has been suggested [44], and in this context, it is noteworthy that we found *CBF1*, which encodes a transcription factor that regulates *MET* genes under the control of Pi conditions and Pho4.

### Pho4 and Stress Response in Yeast

Judging from the Gene Ontology terms of these 18 newly recognized *PHO*-type genes (Saccharomyces Genome Database [SGD], http://www.yeastgenome.org/), they apparently do not have any functional relationship to either Pi metabolism or Pi signaling and are not categorized in a specific functional group (Table S4). Their expression profiles by global analysis, however, showed some similarities in that 12 of them are induced by either nitrogen depletion or amino acid starvation [32,45] and 9 of them are induced in the stationary phase [45]. This raises the possibility that Pho4 is involved in the regulation of a certain set of genes that responds to these nutrient-limiting or stress conditions. Pho4 is also reported to activate the transcription of genes involved in G_1_ arrest caused by DNA damage [28]. Thus, Pho4 appears to activate the transcription of genes responding to various stress conditions. This notion implies possible cross talk between Pi starvation and other stress conditions, the requirement of function of some, if not all, of these 18 genes in the adaptation of yeast cells to Pi starvation, or both.

### Pho4 Can Function Independent of Pi Conditions

The results in this paper imply that Pho4 is present in the nucleus even under high-Pi conditions to activate or repress transcription (Figure 2), an implication that challenges our current understanding of Pho4 regulation. If the current model were correct, then Pho4 should somehow avoid phosphorylation by Pho85, or if phosphorylated, then the modified Pho4 should have much less affinity to the Msn5 exportin to remain in the nucleus. Recently, Zappacosta et al reported Pi-dependent phosphorylation of Pho4 at Ser242 and Ser243 by a kinase other than Pho85 [46]. Phosphorylation of these two sites, however, appears less dependent on Pi than that at those sites modified by Pho85 (i.e., Ser at 100, 114, 128, 152, and 223) [46]. We could imagine that, under high-Pi conditions, prior phosphorylation of the Ser242, Ser243, or both by this unknown kinase could prevent phosphorylation of the other Ser residues by Pho85, thereby decreasing the affinity of Pho4 for Msn5 while increasing the affinity to the target promoter, including *ASN1*. Alternatively, Pho4 modified at Ser242, Ser243, or both might have more affinity to a yet unknown factor than to the exportin, and the resulting complex might be recruited to the target promoter regardless of phosphorylation by Pho85.

The Pho4 transcription factor appeared to repress *CIS3* and *YPS3*, both cell wall constituents. Expression of *CIS3* is repressed by nitrogen starvation and in the stationary phase, implying that Pho4 can function as both an activator and a repressor under these stress conditions. The functioning of a yeast transcription factor as both an activator and a repressor has precedents (e.g., Rap1 and Abf1) [47,48]. Transcriptional repression by the two factors is often accompanied by silent chromatin structure. In a separate paper, we have reported that Pho4 negatively regulates the expression of *SNZ1*, a stationary phase-specific gene, and that this regulation is accompanied by alterations in chromatin structure evoked by Pho4 binding [49]. The mechanism underlying transcriptional repression of *CIS3* and *YPS3* by Pho4 is yet to be clarified, but we suppose that a similar mechanism with the *SNZ1* may apply in these cases.

### Biologically Functional Yeast Antisense and Intragenic RNAs

We demonstrated the presence of Pi-regulated AS RNA in the *KCS1* locus. A large-scale cDNA sequencing by Miura et al. revealed the presence of many AS RNA species [17], including an AS RNA in the *KCS1* locus transcribed from +293 to −43. Although its start point is different a little from our result by 5′-RACE analysis (Figure 3E), we think it highly likely that this AS RNA coincides with the Pi-regulated AS RNA that we have reported here. The Pi-regulated AS RNA in *KCS1*, however, did not appear to be coregulated with the *KCS1* mRNA in the wt cells (Figure 3C), and this contrasts with the observation in higher eukaryotes that sense and AS pairs are frequently coregulated [50]. Although Miura et al. did not describe the regulation of the *KCS1* AS RNA, they claim coregulation between the sense and AS RNA in the *GAL10* locus. However, the fact that the fold induction of the AS RNA is much less compared to that of the *GAL10* mRNA when cells are grown in galactose medium and that Gal4 dependency of the *GAL10* AS RNA was not analyzed points out that more work is necessary to establish coregulation of the sense and AS RNA at the *GAL10* locus.

With respect to biological function of ncRNA in yeast, a noncoding intergenic transcript (*SRG1*), originating from upstream of *SER3* on the same strand and activated by Cha4 transcription factor in the presence of serine [13], inhibits the binding of activators to the *SER3* upstream activating sequence and of TATA-binding protein to its TATA box, leading to repression of *SER3* [12]. Yeast AS RNA has been reported in the *IME4* locus, which is expressed only in the haploid state to inhibit the *IME4* mRNA transcription by transcriptional collision and thereby determines cell fate (i.e., the entry into meiosis) [16]. The AS RNA at the *PHO5* locus is constitutively expressed at a low level from ca. 1,400 bp downstream of the *PHO5* TATA box through its promoter and is proposed to increase chromatin plasticity to enhance histone eviction upon a shift to low-Pi conditions [14]. Those in the *PHO84* locus are suggested to recruit and/or stimulate Hda1 histone deacetylase for silencing of *PHO84* in aging yeast cells [15]. Although these AS RNA species are found in *PHO* genes, they have not been reported to be regulated by environmental Pi conditions to facilitate the activation of *PHO* genes. The *KCS1* case presents a different situation from them in that the AS and intragenic RNAs are activated by Pho4 in response to Pi starvation and may modulate the level of Kcs1 IP_6_ kinase to enhance Pi signaling, thereby stimulating the activation of *PHO* genes. We observed a decrease in the *KCS1* mRNA level when Pho4 binds to the *KCS1* ORF under low-Pi conditions or in a Δ*pho85* mutant (Figure 3C, lanes 2, 5, and 6). This observation suggests a possibility that transcriptional elongation of *KCS1* mRNA is inhibited directly by Pho4 binding within the ORF. However, the scenario may not be so simple, because the *KCS1* mRNA transcription itself can interfere with Pho4 binding, as reported in the *SER3*/*SRG1* case [12]. Alternatively, the AS RNA could cause transcriptional collision with the mRNA, and hybrid formation of the AS RNA with the mRNA could lead to degradation of the mRNA. Both of these events could lead to a reduction in the *KCS1* mRNA level.

The stimulation of low-Pi signaling by Pho4-dependent intragenic and AS RNA represents an autoregulation (induction) or positive feedback loop responding to Pi limitation that can be envisioned as follows. Upon Pi limitation, the low-Pi signal is transmitted to Pho81, leading to inhibition of Pho85-Pho80 and thereby stimulating Pho4 migration into the nucleus [6]. Pho4 then activates transcription of the AS and intragenic RNAs in the *KCS1* locus: the AS RNA could reduce the *KCS1* mRNA level by hybrid formation and possible transcriptional collision, which can lead to stabilization of Pho4 binding, resulting in the production of more AS and intragenic RNAs and consequently of more truncated Kcs1 protein using the downstream ATG codon at +676. These events could lead to down-regulation of Kcs1 activity, enabling Vip1 IP_6_ kinase to utilize more IP_6_ to synthesize 4- or 6-PP-IP_5_ functioning in low-Pi signaling. Because Kcs1 also can phosphorylate these IP_7_ species to synthesize 4,5- or 5,6-PP_2_-IP_5_ (IP_8_) [37], reduction of the Kcs1 level can ensure accumulation of the IP_7_ species to further stimulate low-Pi signaling, leading to complete inhibition of Pho85-Pho80. When Pi becomes sufficient, this loop runs in an opposite way for efficient inactivation of Pho4 and consequent repression of *PHO* genes. Though putative Pho4 binding sequences are present at −464 and −154 in its promoter, *VIP1* expression was dependent on neither Pi condition nor Pho4 (unpublished data), as in the case of the *KCS1* mRNA. Inositol polyphosphate (IP) plays an important role in intracellular signal transduction as second messengers. The absence of Kcs1 and Vip1 causes abnormal vacuolar function and cell morphology, respectively, suggesting that they bear important cellular function [37,51]. Therefore, it is reasonable that the genes involved in IP synthesis are not regulated directly by individual nutrients (in this case, Pi) but indirectly by AS and intragenic RNAs responding to the nutrient, so that signal transduction and normal cellular function are not easily perturbed by fluctuation in the status of an individual nutrient.

Positive feedback in the *PHO* system is also suggested to function in switching of Pi transporters [52], in which Spl2, activated by Pho4, down-regulates low-affinity Pi transporters, Pho87 and Pho90, whereas high-affinity Pi transporter Pho84 is activated by Pho4. When the intracellular Pi level increases via the high-affinity transporter, Pho4 is inactivated to switch the transporters.

### *PHO* System To Explore Intimately Wired Transcriptional Regulation System

Our finding expands the role of the Pho4 transcription factor beyond the regulation of the *PHO* system. The current consensus view is that it is the master regulator of the genes involved in the system, in that Pho4 activates transcription of the structural genes composing the *PHO* system to coordinate cellular response to Pi starvation [3]. Our findings indicate that Pho4 can modulate the activity levels of the products of apparently non-*PHO* genes by activating antisense and intragenic RNA expression to stimulate low-Pi signal transduction. A Δ*pho85* deletion causes pleiotropic mutant phenotypes [1], some of which could be based on otherwise dormant transcriptional initiation, either intergenic or intragenic, or on the AS strand, caused by hyperactive Pho4. In fact, we have also found Pi- and Pho4-regulated AS RNA in the *GTO3* locus and intragenic sense transcript in the *SHE9* locus (unpublished data). *GTO3* encodes an omega class glutathione-S-transferase having glutaredoxin activity, which is suggested to maintain an adequate redox state of specific target proteins, not in the general defense against oxidative stress [53]. *SHE9*, also known as *MDM33*, encodes a mitochondrial inner membrane protein functioning to maintain mitochondrial morphology [54]. Although, at present, we are unable to elucidate whether the *GTO3* AS RNA or the *SHE9* intragenic transcript can affect the annotated function of the respective gene product, this line of work will lead us to uncover yet unknown protein functions in the cellular response to Pi starvation.

Regulation of gene expression and function by these nonconventional RNA species that are regulated by nutrient signals need not be restricted to the *PHO* system. Other inducible systems including *GAL*, glucose repression, and various stresses may well have these RNA species regulated by corresponding signals. High-throughput cDNA sequencing by Miura et al. and other works using microarrays [17–19] have revealed the presence of many intergenic, intragenic, and antisense RNAs in the yeast transcriptome. The finding of nutrient-regulated RNAs that are not coding annotated proteins adds more complexity to the intimately wired transcriptional regulation system by which yeast cells adapt to alterations in environmental conditions. High-resolution mapping of transcription factor and RNA polymerase II binding and very recent development of DNA–RNA hybridization techniques [55] will help to identify these regulatory RNAs. The yeast system, which can be manipulated by an array of genetic tools and for which there exists a substantial body of genetic information, will be the best resource to explore the complexity of the genetic network, including ncRNA species that function in responding to external signals.

## Materials and Methods

### Yeast strains and molecular biology.

Standard yeast genetics and media were used as described [56]. For phosphate-limited medium, Yeast Nitrogen Base (YNB) without phosphate (Q-Biogene) was used instead of normal YNB (SD medium) and was supplemented with 0.2 μM or 2 mM sodium phosphate to make low- and high-Pi media, respectively. The yeast strains used in this work are listed in Table S5.

### DNA manipulation.

Standard Escherichia coli and yeast protocols were employed [56,57]. Plasmids and primers used in this work are listed in Tables S5 and S6, respectively. A Δ*pho85*::*URA3* fragment [25] was used to disrupt the *PHO85* locus of the BY4741 (MFY371) strain, and successful disruption was confirmed by PCR and constitutive expression of acid phosphatase (unpublished data). To disrupt the *PHO4* locus, Pho4Δ-F and -R primers were used to amplify the *LEU2* marker having *PHO4* sequences (from +1 to +100 and from +830 to 929 with A of ATG as +1) at its termini, and the resulting fragment was introduced into MFY371. Successful disruption was confirmed by PCR and failure to express *PHO5*. For disruption of the *VIP1* and *RRP6* loci, the adaptamer-mediated PCR method was employed to prepare the DNA fragments for disruption [58]. The detailed methods are described in Text S1. Disruption of *GCN4* is described elsewhere [49]. To construct *PHO4*-tagged strains, MFY376 and MFY377, a fragment containing *PHO4* tagged with His × 6 and Flag × 3 was amplified using primers Pho4-Flag-F and -R and pUG6H3Flag plasmid as a template [59], followed by transformation of MFY371 and MFY373, respectively. Rpo21 fragments tagged with His × 6 and Flag × 3 (Rpo21-Flag-F and -R) were used to construct MFY378 and MFY379.

To mutagenize the prospective Pho4 binding site in the *ASN1* promoter and in the *KCS1* ORF, a QuickChange II site-directed mutagenesis kit (Stratagene) and appropriate primers were used. The detailed methods are described in Text S1. Successful mutagenesis and the whole sequence of the mutant *ASN1* promoter and of the mutant *KCS1* ORF were confirmed by DNA sequencing. The promoter (−920 to −1) and ORF (−1 to +3143) of *KCS1* were amplified by PCR using MN1132/1133 and MN1134/1135 pairs, respectively, so that EcoRI-NcoI and NcoI-XhoI fragments containing the respective sequences were generated. The two fragments were then ligated through the NcoI site and introduced into pRS313 to generate the pMF1530 plasmid. The wt NcoI-BamHI (+1875) fragment had been replaced by the mutant fragment that lacked the three prospective Pho4 binding sites prior to incorporation of the EcoRI-XhoI *KCS1* fragment into pRS313 to generate the pMF1531 plasmid. To construct plasmids pMF1527 and pMF1529 producing the wt and mutant Kcs1 protein tagged with six copies of the c-*myc* epitope at their C-termini, respectively, the EcoRI-XhoI fragment containing the wt or mutant *KCS1* sequence was introduced into pRS316 containing a 6 × *myc* sequence. Plasmids pMF1540 and pMF1560 overexpressing the *KCS1* AS RNA were constructed by placing the KpnI-EcoRI (+291 to −920) fragment downstream of the *TDH3* and *GAL1* promoters in the pRS323 plasmid, respectively. Plasmid pMF1563 overproducing N-terminally truncated Kcs1 protein (dKcs1) was constructed by placing the NcoI-XhoI fragment (+676 to +3150) that had been cloned by PCR downstream of the *TDH3* promoter in pRS326.

### ChIP-on-chip analysis.

Yeast cells producing tagged protein were cultivated in high- or low-Pi medium as described above to a cell density of *A*_600_ = 1.0–1.2, and chromatin immunoprecipitation was carried out as described [60]. ChIPed fragments were amplified by the T7 RNA polymerase-mediated method (T7RPM) followed by cDNA synthesis and the ligation-mediated PCR (LM-PCR) method for HR and LR analysis, respectively, essentially as described [41,61]. For T7RPM, ChIPed DNA (about 100 ng) was dephosphorylated in the reaction mixture (30 μl) containing 2 units of CIAP and 0.2 units of BAP at 37°C, followed by incubation at 50°C for 15 min each. DNA was purified using a MinElute Reaction CleanUp kit (Qiagen) and eluted from the column with 10 μl of elution buffer (10 mM Tris.HCl, pH8.0). This cleanup method was used throughout the following procedure except for the cleanup of reactions containing RNA. Dephosphorylated DNA was then subjected to poly(dT) tailing reaction in a reaction mixture (20 μl) containing terminal transferase buffer (Roche), 1.25 mM CoCl_2_, 2.5 μM dNTP, and 20 units of terminal transferase by incubating at 37°C for 15 min. T7A18B primer (GCATTAGCGGCCGCGAAATTAATACGACTCACTATAGGGAG[A]18B, where B refers to C, G, or T) was then annealed to the dT-tailed DNA by incubating at 94°C for 2 min, at 35°C for 2 min, and at 25°C, followed by extension reaction in a 50 μl reaction mixture containing 1 ng/μl tailed DNA, 0.5 mM dNTP, 1 unit of Klenow enzyme, and 5 units of Sequenase at 37°C for 60 min. DNA was then subjected to in vitro transcription using a T7 Megascript kit (Ambion) in 20 μl of reaction mixture at 37°C for 4 h, followed by cleanup with an RNEasy Mini kit (Qiagen). One microliter of 100 μM T7 degenerate primer (GGATCCTAATACGACTCACTATAGGAACAGACCACCNNNNNNNNN) was added to the RNA product, which was incubated at 70°C for 10 min, then on ice for 2 min, followed by cDNA synthesis. About 500 ng of cDNA was subjected to a second round of in vitro transcription and subsequent cDNA synthesis, followed by labeling using an in vitro transcription labeling kit (Affymetrix). Labeled cRNA was hybridized to Affymetrix high-density oligonucleotide arrays of S. cerevisiae whole genome (Watson strand) or of chromosomes 3, 4, 5, and 6, which were processed and analyzed as described [62]. For LM-PCR, phosphorylation of 5′ termini of ChIPed DNA fragments by T4 polynucleotide kinase and ATP was performed prior to the blunt-end reaction, followed by ligation of annealed linkers (MN974 and MN975) at 15°C for 16 h. The resulting fragments were amplified by PCR using MN974 primer, followed by PCR labeling with Cy3-dUTP and Cy5-dUTP for ChIPed and whole cell extract DNA, respectively. The data were analyzed with ChIP Analytics 3.0 software (Agilent).

### Analytical methods.

The procedures for RNA isolation, northern and immunoblot analyses, and assay for β-galactosidase were as described [25,43]. DNA probes were prepared by PCR using digoxigenin (DIG)-PCR labeling mix (Roche). RNA probes were prepared by transcribing DNA fragment cloned in pSP72 or pSP73 (Promega) with T7 RNA polymerase and a DIG-RNA labeling mix (Roche). Gene-specific PCR was performed using primers listed in Table S6 and ChIPed DNA fragments or DNA in the whole cell extract (WCE) fraction as template under cycling condition as described [49]. A 5′-Full-RACE kit (TaKaRa) was used to determine the transcription start points in the *KCS1* locus with total RNA from Δ*pho85* cells grown in high-Pi medium. Phosphorylated primers MN915 and MN1190 were used as a reverse transcription primer for the start points upstream of the initiating codon and within the ORF, respectively, and MN1134 for the start point of AS RNA. Amplified fragments were cloned using a TOPO TA cloning kit (Invitrogen) according to the manufacturer's protocol, and the transcription start points were determined by DNA sequencing. RNase protection assay and RT-PCR were carried out essentially as described [57]. Total RNA was digested with RNase ONE (Promega) at 30°C for 1 h and was recovered by precipitation in the presence of ethanol. First-strand cDNA was then synthesized using a primer specific to the sense or antisense strand, followed by PCR amplification after inactivation of reverse transcriptase and addition of appropriate reverse primer. The PCR products were separated by electrophoresis on a 5% polyacrylamide gel.

## Supporting Information

Table S1 Accession Numbers of the Genes Described in This Article (48 KB DOC)

Table S2 Genes That Bind Pho4 Dependent on Phosphate Conditions (32 KB XLS)

Table S3 Genes That Bind Pho4 Independent of Phosphate Conditions (44 KB XLS)

Table S4 Gene Ontology Terms and Expression Profile of Novel *PHO*-Type Genes (52 KB XLS)

Table S5 Yeast Strains and Plasmids Used in This Work (38 KB XLS)

Table S6 Primers Used in This Work (48 KB XLS)

Text S1 Supporting Methods (48 KB DOC)

## Abbreviations

AS RNA: antisense RNA
ChIP: chromatin immunoprecipitation
DIG: digoxigenin
IP: inositol polyphosphate
ncRNA: noncoding RNA
Pi: inorganic phosphate
