# Supplementary material for: Nutrient-Regulated Antisense and Intragenic RNAs Modulate a Signal Transduction Pathway in Yeast
Source: PLoS Biol. 2008 Dec 23;6(12):e326. doi: 10.1371/journal.pbio.0060326 (PMC2605928; doi:10.1371/journal.pbio.0060326)
Supplement: Table S1 — (48 KB DOC) [file pbio.0060326.st001.doc]

Supplementary Table S1. Accession numbers of the genes described in this article.

| Gene | Entrez GeneID |
| --- | --- |
| *ABF1* | 853748 |
| *ARO9* | 856539 |
| *ASN1* | 856268 |
| *CBF1* | 853523 |
| *CDC19* | 851193 |
| *CIS3* | 853282 |
| *CYC3* | 851192 |
| *GAL10* | 852307 |
| *GAL4* | 855828 |
| *GCN4* | 856709 |
| *GFD1* | 855297 |
| *GLN1* | 856147 |
| *GSY2* | 850962 |
| *GTO3* | 855292 |
| *HOM3* | 856778 |
| *HOR7* | 855293 |
| *HPF1* | 854010 |
| *ILV3* | 853473 |
| *IME4* | 852683 |
| *KCS1* | 851580 |
| *MNN1* | 856718 |
| *MUC1* | 854836 |
| *PDC1* | 850733 |
| *PHO4* | 850594 |
| *PHO5* | 852390 |
| *PHO8* | 852092 |
| *PHO80* | 854161 |
| *PHO85* | 856076 |
| *PST1* | 851625 |
| *PTK2* | 853522 |
| *RAP1* | 855505 |
| *RPO21* | 851415 |
| *RRP6* | 854162 |
| *SHE9* | 852002 |
| *SPL2* | 856538 |
| *SUR1* | 856050 |
| *TOR1* | 853529 |
| *UGP1* | 853830 |
| *URA3* | 856692 |
| *VIP1* | 851126 |
| *YPS3* | 850812 |
