# Supplementary material for: Nutrient-Regulated Antisense and Intragenic RNAs Modulate a Signal Transduction Pathway in Yeast
Source: PLoS Biol. 2008 Dec 23;6(12):e326. doi: 10.1371/journal.pbio.0060326 (PMC2605928; doi:10.1371/journal.pbio.0060326)
Supplement: Text S1 — (48 KB DOC) [file pbio.0060326.sd001.doc]

**Supplementary TEXT S1**

**Methods**

**DNA manipulation**

A Δ*pho85*::*URA3* fragment [25] was used to disrupt the *PHO85* locus of BY4741 (MFY371) strain, and successful disruption was confirmed by PCR and constitutive expression of acid phosphatase (data not shown). To disrupt the *PHO4* locus, Pho4Δ-F and -R primers were used to amplify *LEU2* marker having *PHO4* sequences (from +1 to +100 and from +830 to 929 with A of ATG as +1) at its termini, and the resulting fragment was introduced into BY4741. Successful disruption was confirmed by PCR and failure to express *PHO5*.

For disruption of the *VIP1* locus, the adaptamer-mediated PCR method was employed to prepare the DNA fragments for disruption [56]. Two parts of the *VIP1* ORF fragments containing the 5’- (+200 to +880) and 3’- (+1145 to +3400) segments were amplified with MN1141/1144 and MN1145/1142 pairs to place the inverted reverse (IR) tag sequence, GCAGGGATGCGGCCGCTGACG, at its 3’ terminus and the inverted forward tag (IF) sequence, CCGCTGCTAGGCGCGCCGTGGA, at its 5’-terminus, respectively. Two partially overlapping fragments of the *HIS3* gene from *C. grablata* (*CgHIS3*) were amplified with the primer sets MN362/347 and MN346/1066 to place the IR tag at the 5’-terminus, and the IF-tag at 3’-terminus, respectively. The *VIP1* and *CgHIS3* fragments having corresponding tags were combined, annealed through the tag, which were then used as template for PCR with MN1141/347 and MN346/1142 to prepare fusion fragments having 5’- and 3’-portions of *CgHIS3*, respectively. The two PCR fragments were combined and used to transform yeast, and successful disruption of the *VIP1* locus in resulting His^+^ transformants was confirmed by PCR. Disruption of the *RRP6* locus was carried out similarly using MN1195/1196 and MN1197/1198 pairs and *KlURA3* fragments.

To construct *PHO4*-tagged strains, MFY376 and MFY377, a fragment containing *PHO4* tagged with His x 6 and Flag x 3 was amplified using primers Pho4-Flag-F and –R and pUG6H3Flag plasmid as template [59], followed by transformation of BY4741 and MFY373, respectively. Rpo21 fragment tagged with His x 6 and Flag x 3 (Rpo21-Flag-F and -R) were used to construct MFY378 and MFY379. Successful tagging of Pho4 and Rpo21 proteins was confirmed by western analysis using anti-Flag antibody (data not shown).

To mutagenize Pho4 binding site in the *ASN1* promoter, about 1 kbp of the *ASN1* promoter sequence was amplified by PCR with MN1000/1001 primers and inserted into pMF811 plasmid [25] to construct pMF1486. The prospective Pho4 binding site in the *ASN1* promoter (at -451) was mutagenized using a QuickChange II site-directed mutagenesis kit (Stratagene) and MN929/930 primers. Successful mutagenesis and the whole sequence of the *ASN1* promoter were confirmed by DNA sequencing. To mutagenize three prospective Pho4 binding sites in the N-terminal half of *KCS1* ORF (+406, +1127, and +1393), an *Nco*I (-1) – *Bam*HI (+1875) fragment containing the three sites and used as template for mutagenesis was derived from a *KCS1* fragment amplified by PCR using MN1134/1135 primers to incorporate *Nco*I and *Xho*I at its 5’- and 3’-temini, respectively. The three sites were mutagenized successively using a QuickChange II kit and MN952/953, MN1136/1137, and MN1138/1139 primer pairs. Successful mutagenesis and the whole sequence of the N-terminal half of the *KCS1* ORF were confirmed by DNA sequencing. The promoter (-920 to -1) and ORF (-1 to +3143) of *KCS1* were amplified by PCR using MN1132/1133 and MN1134/1135 pairs, respectively, so that *Eco*RI-*Nco*I and *Nco*I-*Xho*I fragments containing respective sequence were generated. The two fragments were then ligated through the *Nco*I site and introduced in to pRS313 to generate pMF1530 plasmid. The wt *Nco*I-*Bam*HI (+1875) fragment had been replaced by the mutant fragment that lacked the three prospective Pho4 binding sites prior to incorporation of the *Eco*RI-*Xho*I *KCS1* fragment into pRS313 to generate pMF1531 plasmid. To construct plasmids pMF1527 and pMF1529 producing the wt and mutant Kcs1 protein tagged with 6 copies of c-myc epitope, respectively, the *Eco*RI-*Xho*I fragment containing the wt or mutant *KCS1* sequence was introduced into pRS316 containing a 6 x myc sequence. Plasmid pMF1560 overexpressing the *KCS1* AS RNA was constructed by placing the *Kpn*I-*Eco*RI (+291 to -920) fragment downstream of the *GAL1* promoter in pRS323 plasmid.
